# First Results Comparing MLC Versus IRIS CyberKnife Collimators in Prostate Stereotactic Body Radiation Therapy in an Italian Oncology Institute

**DOI:** 10.3390/bioengineering13060596

**Published:** 2026-05-22

**Authors:** Gaetano Gagliardo, Marcello Serra, Gianluca Ametrano, Rosario Megna, Valentina d’Alesio, Francesca Buonanno, Cecilia Arrichiello, Rossella Di Franco, Valentina Borzillo, Esmeralda Scipilliti, Rocco Mottareale, Simona Mercogliano, Mariagabriella Pugliese, Maria Quarto, Vincenzo Ravo, Paolo Muto

**Affiliations:** 1 Post Graduate School in Medical Physics, University of Naples Federico II, 80131 Naples, Italy; ga.gagliardo@studenti.unina.it (G.G.); r.mottareale@studenti.unina.it (R.M.); maria.quarto@unina.it (M.Q.); 2 Department of Radiation Oncology, Istituto Nazionale Tumori—IRCCS—Fondazione G. Pascale, 80131 Naples, Italy; marcello.serra@istitutotumori.na.it (M.S.); gianluca.ametrano@istitutotumori.na.it (G.A.); valentina.dalesio@istitutotumori.na.it (V.d.); francesca.buonanno@istitutotumori.na.it (F.B.); c.arrichiello@istitutotumori.na.it (C.A.); r.difranco@istitutotumori.na.it (R.D.F.); valentina.borzillo@istitutotumori.na.it (V.B.); esmeralda.scipilliti@istitutotumori.na.it (E.S.); simona.mercogliano@istitutotumori.na.it (S.M.); v.ravo@istitutotumori.na.it (V.R.); 3 Institute of Biostructure and Bioimaging, National Research Council, 80145 Naples, Italy; 4 Department of Physics “E. Pancini”, University of Naples Federico II, 80126 Naples, Italy; mariagabriella.pugliese@unina.it; 5 Fondazione Muto ETS, 80132 Naples, Italy; p.muto@istitutotumori.na.it

**Keywords:** prostate cancer, CyberKnife, Stereotactic Body Radiation Therapy, MLC, IRIS

## Abstract

Prostate cancer (PCa) is one of the most common malignancies in men and remains a major cause of cancer-related death worldwide. Radiotherapy is a well-established treatment modality for PCa, offering clinical outcomes comparable to surgical approaches. In recent years, stereotactic body radiotherapy (SBRT), characterized by the delivery of high radiation doses in a limited number of fractions, has been increasingly adopted as a standard approach in the treatment of prostate cancer, due to its favorable efficacy and toxicity profile. CyberKnife (CK) is one of the most commonly used hypofractionated radiotherapy techniques. This preliminary study aimed to evaluate and compare the radiation dose delivery and treatment time of CK-based SBRT using two different collimation systems: the multileaf collimator (MLC) and the IRIS variable aperture collimator, a dynamic device that adjusts its opening to simulate different circular field sizes. A total of 19 patients with low-to-intermediate-risk PCa were selected and treated at the Radiation Oncology Department of the National Cancer Institute IRCCS Fondazione G. Pascale in Naples between January 2024 and January 2025. For each patient, two treatment plans were generated—one with the IRIS collimator and one with the MLC. The results demonstrated that the use of the MLC significantly reduced treatment time while maintaining dosimetric quality comparable to IRIS-based plans. These findings support the clinical benefit of MLC implementation in prostate SBRT with the CK system.

## 1. Introduction

Prostate cancer (PCa) is one of the most prevalent malignancies in men and represents a leading cause of cancer-related mortality worldwide [1]. Its incidence increases with age, from 5% in individuals under 30 to 59% in those over 79 (odds ratio per decade: 1.7) [2]. Due to population aging, annual cases are expected to reach 2.3 million by 2040 [3]. Incidence is higher in Western countries, likely due to genetic and screening factors [4]. In 2022, the US reported 230,125 new cases (age-standardized rate (ASR): 75.2), Germany 65,269 (ASR: 54.2), the UK 55,485 (ASR: 74.0), and France 57,357 (ASR: 82.3) [5]. In Italy, 41,100 new cases were diagnosed in 2023, with a rise of 18% since 2017 [6].

Radiotherapy is a well-established modality for managing PCa [7], achieving outcomes comparable to those of surgical interventions [8,9]. The choice of the optimal treatment strategy is guided by the tumor risk stratification, the patient’s overall health status, individual preferences, and assessment of potential side effects [10]. Specifically, the Stereotactic Body Radiation Therapy (SBRT) is a technique that delivers high-dose, highly precise radiation in fewer sessions than conventional radiotherapy and has become a leading approach for the radiotherapy management of localized PCa, particularly in cases with low-to-intermediate-risk profiles [11,12].

Recent advancements in radiotherapy techniques, especially SBRT, have significantly contributed to the enhanced accuracy and efficacy of treatment delivery in PCa cases [13]. Additionally, emerging evidence suggests that personalized treatment regimens, tailored based on patient-specific factors, further optimize outcomes.

Hypofractionated radiotherapy is a treatment regimen in which radiation is administered in fewer fractions with higher doses per fraction compared to conventional fractionation, maintaining therapeutic efficacy and reducing the overall duration of treatment. Among hypofractionated radiotherapy techniques, the CyberKnife (CK) system stands out for its capacity to deliver highly conformal dose distributions with steep gradients, utilizing a linear accelerator mounted on a robotic arm and guided by stereoscopic imaging [14]. CK delivers the dose with very high precision, as evidenced by the reduction in the Planning Target Volume (PTV) margins. However, this precision presents challenges, such as longer treatment times and potential inefficiencies associated with the sequential beam positioning required by the robotic arm of the CK system [15]. In fact, CK treatment protocols require a large number of monitor units (MUs) and long treatment times due to the use of numerous small, collimated circular radiation beams. These beams are delivered sequentially as the robotic arm transitions between multiple non-coplanar beam positions to achieve optimal target coverage.

Evidence from the literature [16,17], as well as our clinical experience [18,19,20,21,22], indicate that the choice of a collimator significantly impacts both the number of MUs and the treatment duration in SBRT for PCa. Therefore, investigating alternative collimation systems could provide valuable insights into improving treatment efficiency and patient comfort [23,24].

This exploratory study aims to evaluate and compare radiation dose delivery, dosimetric plan quality, and treatment time when using MLC versus IRIS collimators for prostate SBRT. Key parameters under investigation include dose distribution, adjacent organs at risk (OAR) sparing, and beam-on time. Typically, the delivered MUs are also considered among key parameters. However, in this study, given that both treatment plans prescribe the identical radiation dose with the same optimization objectives, the beam-on time and total MUs are highly correlated. Therefore, we focused our primary efficiency analysis on treatment duration, which most directly impacts patient comfort and intra-fraction motion.

## 2. Materials and Methods

### 2.1. The Linear Quadratic Model

In the linear quadratic model, widely used to describe cellular response to ionizing radiation, the parameters α and β represent two components of radiation-induced damage: α [1/Gy] corresponds to damage resulting from single-track (linear) events proportional to the dose, whereas β [1/Gy^2^] accounts for damage due to the interaction of sub-lethal events, inversely proportional to the square of the dose [25]. The α/β [Gy] ratio reflects the tissue’s sensitivity to fractionation; indeed, differences in α/β ratios between the tumor and surrounding healthy tissue play a key role in determining the fractionation regimen: Tumor Control Probability (TCP) and Normal Tissue Complication Probability. Due to the pronounced variation in α/β ratios between prostate tumors and adjacent organs at risk (OARs), hypofractionation is strongly recommended in radiotherapy of PCa [26,27]. Tissues with low α/β values, like prostate tumors (typically ~1.5 Gy), benefit more from hypofractionation, as larger doses per fraction increase tumor control while sparing normal tissues, which usually have higher α/β values. Therefore, delivery systems that enable sharp dose gradients and high spatial precision—such as the MLC—may be better suited to exploit this radiobiological advantage compared to IRIS, potentially improving the therapeutic ratio.

### 2.2. CyberKnife

**Figure 1:**
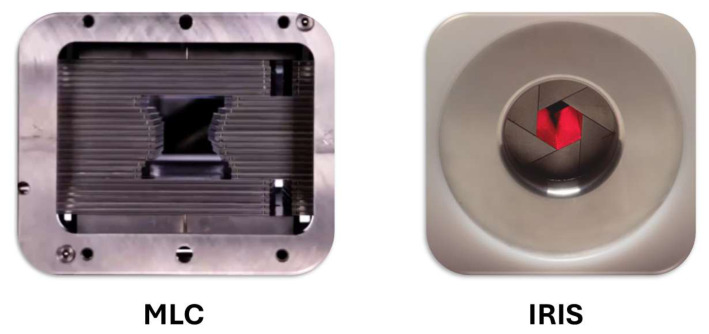
Frontal view of MLC and IRIS collimators [34]. The main difference between collimators lies in the way the beams are delivered: the MLC uses linear leaves, while the IRIS collimator uses a dynamic pseudo-circular aperture.

The CyberKnife (CK, Accuray Inc., Sunnyvale, CA, USA) is a 6 MeV linear accelerator mounted on a robotic arm with 6 degrees of freedom, enabling a non-isocentric approach to dose delivery [28]. The system is equipped with two interchangeable collimation devices: the IRIS variable aperture collimator and the InCise™ multileaf collimator (MLC), both fully integrated into the same platform and producted by Accuray Inc., Sunnyvale, CA, USA. These collimators can be selected alternatively based on clinical needs, offering different approaches to beam shaping while utilizing the same delivery system. Specifically, the CK features static circular collimators with diameters ranging from 5 to 60 mm, along with the dynamic IRIS™ collimator, which consists of two hexagonal diaphragms designed to generate beam profiles that closely approximate circular shapes, matching the nominal field sizes of fixed collimators [29]. The MLC [30] is composed of 41 pairs of leaves, each measuring 2.5 mm in width, providing a maximum field size of 12.00 cm × 10.25 cm. This advanced technology enables dynamic field shaping, allowing for precise adaptation to the tumor’s contours. A graphical representation of the MLC and IRIS collimators is shown in Figure 1.

The system can position the accelerator in 100 distinct nodes, each supporting up to 12 beam orientations, resulting in a total of 1200 possible beam entry angles. These nodes correspond to pre-defined spatial positions around the patient from which the accelerator can irradiate. At each node, the robotic arm allows for multiple beam orientations thanks to its six degrees of freedom, enabling non-isocentric, non-coplanar delivery [31,32]. This setup maximizes dose conformity and minimizes exposure to surrounding healthy tissues, which is particularly advantageous in SBRT. A key advantage of the CK in radiotherapy is its capability to deliver up to 125% (ablative dose) of the prescribed dose within the tumor volume while ensuring significant sparing of the surrounding OARs [33].

The CK utilizes advanced assistive and adaptive technology through image-guided radiotherapy (IGRT). Its integrated imaging system facilitates accurate patient positioning and allows for real-time tracking of target motion throughout the treatment process.

The CK imaging system consists of two X-ray tubes installed on the ceiling, oriented at 90 degrees to each other and tilted 45 degrees to the patient’s axis. Corresponding silicon flat-panel detectors are placed on the floor near the treatment couch. The system operates with a nominal tube voltage ranging between 40 keV and 150 keV. Imaging is performed every 45 or 60 s, generating real-time digital images that are then compared to digitally reconstructed radiographs derived from the patient’s CT scans. This technique enables the identification of intra-fractional target movements and allows the robotic manipulator to automatically adjust the treatment delivery accordingly [33].

In the treatment of PCa, four gold fiducial markers are implanted into the prostate wall to precisely localize the tumor and track positional variations during therapy. These fiducials facilitate the real-time determination of the prostate’s position, allowing the accelerator to dynamically adjust its alignment with the target throughout the treatment [35].

The CK system is specifically optimized for SBRT and has demonstrated favorable outcomes in the treatment of PCa [36,37,38].

### 2.3. Case Selections and Treatment Planning

A total of 19 patients diagnosed with PCa, classified as low–intermediate risk, were selected and treated at the Radiation Oncology Department of the National Cancer Institute IRCCS Fondazione G. Pascale in Naples, between January 2024 and January 2025. Cases were selected to include both the prostate alone and the prostate with seminal vesicles. The patients’ ages ranged from 60 to 88 years, with a median age of 75 years. The median PTV was (113 ± 10) cm^3^. Patient enrolment was based on risk stratification according to the D’Amico classification system [39]. Patients classified as low risk exhibited a clinical stage of T2a or lower, a prostate-specific antigen (PSA) level of ≤10 ng/mL, and a Gleason score of ≤6. In contrast, intermediate-risk patients were identified by a clinical stage of T2b–T2c, a PSA level of ≤20 ng/mL, and a Gleason score of 7 (3 + 4) [40]. Patients presenting a Gleason score of 7 (4 + 3) were not considered for inclusion in the study. The treatment protocol permitted a maximum prostate volume of 90 cc. Although androgen deprivation therapy (ADT) was not routinely included, some patients underwent ADT for up to three months following urological recommendations. ADT was not continued during or after radiotherapy. For staging purposes, multiparametric magnetic resonance imaging (MRI) of the prostate was conducted. In cases where MRI was contraindicated, pelvic computed tomography (CT) was used as an alternative. To finalize the Tumor, Node and Metastasis (TNM) staging, imaging assessments included abdominal and chest CT scans alongside bone scintigraphy.

Target volume delineation was performed based on simulation CT imaging co-registered with fused prostate MRI data. In patients with low-risk disease, the gross tumor volume (GTV) was limited to the prostate gland. For intermediate-risk patients, the GTV included both the prostate and the proximal 2 cm of the seminal vesicles. The clinical target volume (CTV) was considered equivalent to the GTV. To generate the Planning Target Volume (PTV), the CTV was expanded by 3 mm in the posterior direction and by 5 mm in all other directions. The OARs, including the rectum, bladder, penile bulb, femoral heads, bowel, testicles, and neurovascular bundles, were delineated with high precision.

The PTV prescription dose was set at 3625 cGy across five fractions. For each patient, two separate treatment plans were created: one incorporating the IRIS collimator and the other one utilizing the MLC. Treatment planning was performed in agreement by two experienced medical physicists using Accuray’s specialized “VOLO” system, in conjunction with the high-resolution “Ray Tracing” algorithm. Specifically, each plan was developed by a physicist and reviewed by another physicist, who verified the accuracy and safety of the treatment plan. This procedure helps define the generalizability of the results. To facilitate a reliable dosimetric comparison, both plans were designed to ensure identical PTV coverage, regardless of the collimator type. PTV coverage was maintained between 90% and 100%, with a median value of 96%. All treatment plans, using both collimator types, were designed in compliance with the dose–volume constraints outlined in Table 1 to ensure their acceptability [41]. Subsequently, experienced radiation oncologists thoroughly reviewed each plan to confirm its clinical feasibility.

### 2.4. Comparative Dosimetric Analysis and Statistical Evaluation

To assess treatment quality, the following indicators were evaluated:

- Conformity Index (CI);
- New Conformity Index (nCI);
- Homogeneity Index (HI);
- Gradient Index (GI);
- Dose to the target and OARs;
- Treatment duration.

The Conformity Index (CI) evaluates how well the dose distribution conforms to the target volume. In this study, we utilized the definition from [42], where CI is calculated as the ratio between the total tissue volume receiving the prescribed dose and the target volume that receives the same dose, as described in the following equation:

(1) $$CI=\frac{PIV}{TIV}\;\;,$$

where PIV (Prescription Isodose Volume) represents the entire volume encompassed by the prescribed isodose, while TIV (Target Isodose Volume) refers specifically to the portion of the target that falls within this prescribed isodose boundary.

The new Conformity Index (nCI) quantifies how accurately the prescribed isodose volume matches the shape and size of the target volume [43]. It is essentially a CI normalized for target coverage, as defined in the following equation:

(2) $$nCI=\frac{PTV\times PIV}{{TIV}^{2}}=\frac{CI}{coverage}\;.$$

**Figure 2:**
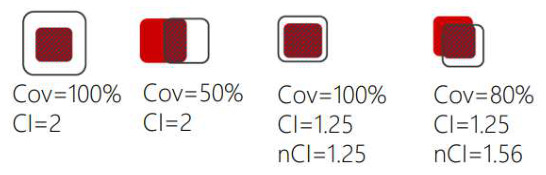
Graphical representation of the Conformity Index (CI) and the new Conformity Index (nCI), and their relationship with target coverage (Cov). The solid red square represents the Target Volume (TIV), while the transparent square with the black border represents the Prescription Isodose Volume (PIV). The intersection between the two shapes illustrates how variations in target coverage affect the nCI value compared to the traditional CI [34].

An illustrative example highlighting the differences between CI and nCI, as well as their relationship with target coverage (Cov), is presented in Figure 2.

The Homogeneity Index (HI) evaluates the consistency of dose distribution within the target. However, due to the intrinsically heterogeneous nature of dose delivery in stereotactic treatments, a modified version of HI was applied for statistical analysis [44,45]. This adapted index specifically assesses the peak dose within the PTV, as detailed in the following equation:

(3) $$HI=\frac{Dmax}{{RX}_{dose}}\;,$$

where Dmax represents the highest dose delivered to the PTV, while RX_dose_ corresponds to the prescribed dose for the target volume.

The Gradient Index (GI) characterizes the dose fall-off rate in stereotactic treatments. As described in [46,47], it is calculated as the ratio between the volume enclosed by a specific reference isodose (PIV%) and the Prescription Isodose Volume (PIV), as presented in the following equation:

(4) $$GI\%=\frac{{PIV}_{\%}}{PIV}\;.$$

For example, PIV_50_ is determined representing the volume encompassed by the 50% isodose level relative to the prescribed dose. The GI at 50% is then calculated as GI50 = PIV_50_/PIV.

### 2.5. Statistical Analysis

Variables were expressed as median and range, and mean ± standard deviation (SD). Normality of the data distributions was verified using the Shapiro–Wilk test. To quantify the performance of collimators, we calculated the percentage variation with respect to the MLC of position parameters between analogous variables, as appropriate. To evaluate the relationship between the data obtained through the MLC and IRIS collimators, we assessed both the statistical difference and correlation between pairs of variables. Statistical difference between data distributions was assessed using the paired *t*-tests or Wilcoxon matched-pairs signed-rank test, as appropriate. Statistical correlation between the pairs of variables was assessed by the Pearson’s coefficient (r) or Spearman’s coefficient (ρ), as appropriate. We considered as highly or moderately correlated pairs of variables with the correlation coefficient respectively greater than or equal to 0.70, and between 0.50 and 0.70. Two-sided *p*-values <0.05 were considered statistically significant. Statistical analysis was performed using the R software, version 4.3.1 (The R Foundation for Statistical Software, Vienna, Austria).

## 3. Results

Our cohort consisted of 19 patients aged 60 to 88 years (median: 75 years).

**Figure 3:**
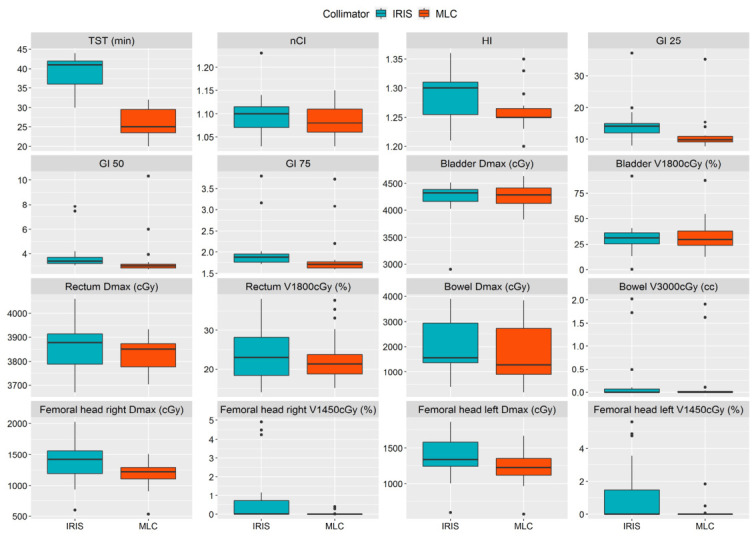
Boxplot comparison illustrating the range and median values of key variables obtained with MLC and IRIS collimators. Dots represent outliers outside the 95% confidence interval.

Figure 3 shows a graphical comparison of range and median values of the variables calculated by the MLC and IRIS collimators.

**Figure 4:**
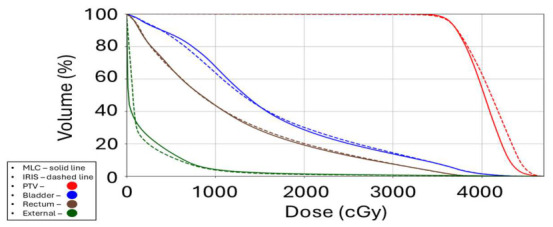
Comparison between dose–volume histograms obtained by the MLC and IRIS collimators. In the graph are reported mean values for the PTV, bladder, rectum, and external, defined as the body excluding all critical regions except the PTV.

Generally, the distributions obtained with the MLC show lower values than those obtained with the IRIS collimator. Figure 4 shows the dose–volume histogram containing the mean values for the PTV, bladder, rectum, and external, defined as the body excluding all critical regions except the PTV.

**Figure 5:**
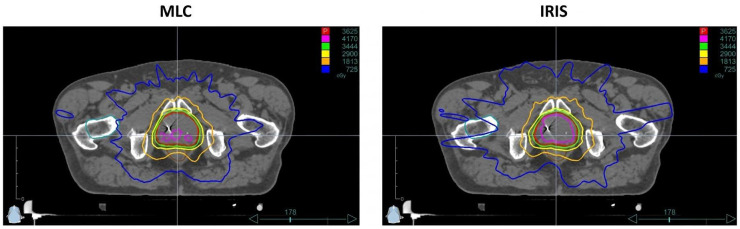
Isodose distributions for MLC and IRIS collimators on the same CT slice of a representative patient. Dose levels are shown at 100% (red), 115% (magenta), 95% (green), 80% (yellow), 50% (orange), and 20% (blue) of the prescribed dose. IRIS shows a broader high-dose spread, while MLC results in a more confined low-dose region.

Figure 5 shows the isodose distributions on the same CT slice of a representative patient, comparing the MLC and IRIS collimators. The figure shows dose levels corresponding to several percentages of the prescribed dose. It is evident that the IRIS collimator results in a wider dose spread, particularly noticeable in the 115% isodose curve. In contrast, the MLC demonstrates a more confined low-dose bath, as shown by the more restricted 20% isodose.

Table 2 summarizes position (median and mean) and dispersion (range and SD) parameters related to variables obtained through the MLC and IRIS collimators, the percentage variation between position parameters of pairs of variables, and the agreement between analogous variables assessed by statistically significant difference and correlation coefficient.

Overall, 12 of the 16 variables show statistically significant differences across the two collimators. Of the 12 pairs of variables with statistical differences, nine show a significantly high or moderate correlation. Therefore, three pairs of variables were in disagreement for both statistical tests (HI, Femoral head right V1450cGy, and Femoral head left V1450cGy).

## 4. Discussion and Conclusions

In this study, we compared the performance of the MLC and IRIS collimators integrated in the CyberKnife radiotherapy system, and applied in prostate SBRT. Initial results of our analysis indicate a better performance of MLC than IRIS collimators. In particular, the reduction of approximately 33% in the TST allows for a better quality of treatment and improved patient well-being. Slightly lower nCI and HI values indicate greater precision in dose distribution and peak dose within the PTV. The three reduced GI values, and in particular that of GI 25 with a reduction of 22.8%, confirm the greater accuracy of the MLC treatment with respect to the dose drop rate in stereotactic treatments. As a consequence of this increased precision in dose delivery, we observed a greater amount of healthy tissue spared from radiation in the body districts affected by the treatment, with the exception of the bladder. A significant correlation between pairs of different variables indicates a relationship between them, with MLC values lower than IRIS interpretable as a distribution shift. This shift suggests that the use of the MLC not only reduces treatment time and improves dose distribution quality but also induces a consistent effect across multiple dosimetric parameters. This highlights the overall efficiency of the MLC system in treatment planning and delivery, making it a potentially preferable option for prostate SBRT in terms of dosimetric quality, OARs sparing, and delivery efficiency.

These improvements may also translate into reduced intra-fraction motion, due to shorter beam-on times. This aspect is particularly relevant in prostate SBRT, where organ motion can significantly impact dose accuracy. Although not directly evaluated in our study, this hypothesis is supported by the current literature [48,49,50] and may be the subject of future investigation.

The findings of this study qualitatively align with earlier research [51] demonstrating that the implementation of the MLC facilitates a notable decrease in treatment duration, while maintaining comparable standards of plan quality. More specifically, in our study, the TST was 26 ± 4 min using the MLC, with a range of 20 to 32 min, and this result is in line with several previous studies: 29 min, 32 min in [51], and 22 min.

The percentage reduction in TST from the IRIS to MLC observed in our study was −33.2%, closely matching the value reported (−35.6%). However, this reduction is more pronounced than that reported, where the decrease was only −15%. Unlike other studies, the doses were not normalized to ensure equivalent PTV coverage at the prescribed dose for both IRIS and MLC solutions. Regardless, MLC plans demonstrated the most pronounced dose gradient, leading to a significant reduction in GI values, a finding that aligns with the results observed in most published studies [51].

The ability of MLCs to dynamically conform the beam shape to complex target geometries may also offer additional clinical benefits in patients with irregular prostate anatomy or proximity to critical structures, such as in post-surgical or re-irradiation scenarios. This level of adaptability, not achievable with fixed circular collimators like IRIS, may help further reduce the probability of radiation-induced toxicities.

Our study has limitations. First, this is an in silico comparative planning study; therefore, it evaluates dosimetric and technical parameters rather than comparative clinical outcomes (such as PSA response or late toxicity), which cannot be assessed since each patient logically received only one of the two actual treatments. The most important limitation of this study is the small cohort size (19 patients), which restricts the generalizability of the conclusions. To address this, a post hoc power analysis was performed. The analysis confirmed a high statistical power (0.99) for the primary endpoint— the reduction in TST—indicating that the sample size is adequate to support this specific finding. However, for secondary dosimetric variables (such as nCI, Bowel Dmax, and Femoral heads Dmax), the statistical power was sub-optimal (ranging from 0.22 to 0.72). Based on a medium effect size (0.5) and a target power of 0.80, a theoretical sample size of at least 33 patients would be required to robustly confirm these secondary findings. Therefore, these dosimetric results should be considered preliminary and need confirmation in larger cohorts. Another limitation may be due to potential biases introduced by the medical physicist in developing a treatment plan. To mitigate these biases, our plans were obtained with the agreement of two experienced medical physicists.

In this context, as a future perspective we have a revision of this analysis with approximately double the number of patients. Additionally, future studies could benefit from a prospective design, the inclusion of patient-reported outcomes (e.g., quality of life or acute toxicity rates), and longer follow-up periods to assess potential correlations between dosimetric parameters and late toxicity or biochemical control [52,53,54,55]. Incorporating radiobiological modeling could also enhance the predictive value of plan comparisons in terms of TCP and NTCP [56,57].

In conclusion, while exploratory in nature due to the limited sample size, the comparison presented in this study has characteristics of innovation in the field of radiotherapy. In fact, the MLC, developed successively to the IRIS collimator, has demonstrated better performance in prostate SBRT.

The integration of the MLC in CyberKnife systems represents a substantial advancement, not only in terms of dosimetric metrics but also in workflow optimization and patient experience. Considering the increasing clinical demand for high-precision, hypofractionated treatments in PCa, MLC-guided SBRT appears to be a promising standard-of-care evolution in image-guided robotic radiotherapy.

## Figures and Tables

**Table 1:** Dosimetric and volumetric constraints used for treatment plan assessment.

Structure	Metric	Objective
Bladder	DMax (cGy)	<4180 cGy
	D1cc (cGy)	<3800 cGy
	V3750 cGy (cc)	<5 cc
	V3700 cGy (cc)	<10 cc
	V1800 cGy (cc)	<15 cc
	V1800 cGy (%)	<40%
	V3625 cGy (%)	<10%
Rectum	DMax (cGy)	<3800 cGy
	V3600 cGy (cc)	<1 cc
	V2500 cGy (cc)	<20 cc
	V3625 cGy (%)	<5%
	V3260 cGy (%)	<10%
	V2900 cGy (%)	<20%
	V1800 cGy (%)	<50%
Bowel	DMax (cGy)	<2850 cGy
	V3000 cGy (cc)	<1 cc
Femoral head	DMax (cGy)	<2400 cGy
	V1450 cGy (%)	<5%
Penis bulb	V2950 cGy (%)	<50%
Testes	V200 cGy (%)	<20%

**Table 2:** Summary of position (median, mean) and dispersion (range, SD) parameters for variables measured with MLC and IRIS collimators, including percentage variation, statistical difference (*p*-value), and correlation (coefficient) between corresponding variables.

	IRIS		MLC		Variation ^§^	Difference	Correlation
Variable	Median (Range)	Mean (±SD)	Median (Range)	Mean (±SD)	%	*p*-Value	Coeff. (*p*-Value)
TST (min)	41 (30, 44)	39.16 (±4.15)	25 (20, 32)	26.11 (±3.75)	−33.2	**<0.001 ^T^**	**0.612 (0.005) ^P^**
nCI	1.10 (1.03, 1.23)	1.10 (±0.05)	1.08 (1.03, 1.15)	1.08 (±0.03)	−1.4	**0.006 ^T^**	**0.900 (<0.001) ^P^**
HI	1.30 (1.21, 1.36)	1.29 (±0.04)	1.25 (1.20, 1.35)	1.26 (±0.04)	−1.5	**0.024 ^W^**	0.377 (0.111) ^S^
GI 25	14.04 (8.00, 37.10)	14.74 (±6.21)	9.77 (7.73, 35.18)	11.41 (±6.04)	−22.8	**<0.001 ^W^**	**0.832 (<0.001) ^S^**
GI 50	3.39 (3.07, 7.85)	3.89 (±1.36)	3.03 (2.75, 10.31)	3.56 (±1.79)	−12.7	**0.003 ^W^**	**0.914 (<0.001) ^S^**
GI 75	1.88 (1.72, 3.79)	2.01 (±0.53)	1.71 (1.59, 3.72)	1.89 (±0.56)	−8.1	**0.014 ^W^**	**0.937 (<0.001) ^S^**
Bladder Dmax (cGy)	4324 (2907, 4516)	4224 (±347)	4288 (3832, 4635)	4271 (±208)	0.5	0.891 ^W^	−0.001 (0.997) ^S^
Bladder V1800cGy (%)	31.32 (0.32, 91.72)	31.90 (±17.51)	29.61 (12.43, 87.39)	33.64 (±16.09)	−5.5	0.418 ^W^	**0.628 (0.005) ^S^**
Rectum Dmax (cGy)	3877 (3671, 4059)	3863 (±99)	3850 (3704, 3934)	3827 (±70)	−0.9	0.229 ^T^	−0.032 (0.897) ^P^
Rectum V1800cGy (%)	23.01 (14.04, 38.05)	24.52 (±7.59)	21.35 (15.12, 37.68)	22.93 (±6.58)	−9.3	**0.023 ^W^**	**0.851 (<0.001) ^S^**
Bowel Dmax (cGy)	1566 (407, 3895)	2044 (±1022)	1289 (196, 3840)	1742 (±1108)	−20.8	**<0.001 ^T^**	**0.986 (<0.001) ^P^**
Bowel V3000cGy (cc)	0.00 (0.00, 2.02)	0.24 (±0.59)	0.00 (0.00, 1.91)	0.20 (±0.56)	-	0.093 ^W^	**0.890 (<0.001) ^S^**
Femoral head right Dmax (cGy)	1421 (600, 2024)	1364 (±325)	1223 (534, 1507)	1190 (±224)	−11.0	**0.002 ^T^**	**0.753 (<0.001) ^P^**
Femoral head right V1450cGy (%)	0.01 (0.00, 4.91)	0.89 (±1.66)	0.00 (0.00, 0.40)	0.037 (±0.11)	-	**0.006 ^W^**	0.382 (0.106) ^S^
Femoral head left Dmax (cGy)	1334 (593, 1857)	1368 (±299)	1224 (570, 1666)	1216 (±229)	−9.4	**0.010 ^T^**	**0.646 (0.003) ^P^**
Femoral head left V1450cGy (%)	0.00 (0.00, 5.57)	1.15 (±1.96)	0.00 (0.00, 1.84)	0.13 (±0.43)	-	**0.018 ^W^**	0.271 (0.263) ^S^

^§^ Percentage variation of the variable pairs refers to the MLC; ^T^ paired *t*-test; ^W^ Wilcoxon matched-pairs signed-rank test; ^P^ Pearson’s correlation; ^S^ Spearman’s correlation; significant tests are highlighted in bold.

## Data Availability

The raw data are available at https://doi.org/10.5281/zenodo.18833786 (last accessed: 21 May 2026).

## Abbreviations

The following abbreviations are used in this manuscript:

PCa	Prostate Cancer
SBRT	Stereotactic Body Radiation Therapy
CK	CyberKnife
MLC	Multileaf Collimator
IRIS	Iris Variable Aperture Collimator
ASR	Age-Standardized Rate
PSA	Prostate-Specific Antigen
TCP	Tumor Control Probability
OAR	Organ At Risk
PTV	Planning Target Volume
MU	Monitor Units
IGRT	Image-Guided Radiation Therapy
ADT	Androgen Deprivation Therapy
CT	Computed Tomography
CTV	Clinical Target Volume
GTV	Gross Tumor Volume
CI	Conformity Index
nCI	new Conformity Index
HI	Homogeneity Index
GI	Gradient Index
PIV	Prescription Isodose Volume
TIV	Tumor Isodose Volume
